# Natural killer cells as a therapeutic tool for infectious diseases – current status and future perspectives

**DOI:** 10.18632/oncotarget.25058

**Published:** 2018-04-17

**Authors:** Stanislaw Schmidt, Lars Tramsen, Bushra Rais, Evelyn Ullrich, Thomas Lehrnbecher

**Affiliations:** ^1^ Division for Pediatric Hematology and Oncology, Johann Wolfgang Goethe University, Frankfurt, Germany; ^2^ Division of Stem Cell Transplantation and Immunology, Laboratory for Cellular Immunology, Hospital for Children and Adolescents, Johann Wolfgang Goethe University, Frankfurt, Germany; ^3^ LOEWE Center for Cell and Gene Therapy, Cellular Immunology, Johann Wolfgang Goethe University, Frankfurt, Germany

**Keywords:** natural killer cell, adoptive immunotherapy, virus, bacterium, fungus

## Abstract

Natural Killer (NK) cells are involved in the host immune response against infections due to viral, bacterial and fungal pathogens, all of which are a significant cause of morbidity and mortality in immunocompromised patients. Since the recovery of the immune system has a major impact on the outcome of an infectious complication, there is major interest in strengthening the host response in immunocompromised patients, either by using cytokines or growth factors or by adoptive cellular therapies transfusing immune cells such as granulocytes or pathogen-specific T-cells. To date, relatively little is known about the potential of adoptively transferring NK cells in immunocompromised patients with infectious complications, although the anti-cancer property of NK cells is already being investigated in the clinical setting. This review will focus on the antimicrobial properties of NK cells and the current standing and future perspectives of generating and using NK cells as immunotherapy in patients with infectious complications, an approach which is promising and might have an important clinical impact in the future.

## INTRODUCTION

Immunocompromised patients, such as patients suffering from cancer or hematopoietic stem cell transplant (HSCT) recipients, are at a significantly increased risk for an infectious complication due to viral, bacterial or fungal pathogens [1–4]. In addition, in this patient population, infections often have a more severe clinical course and are an important cause of mortality [4]. Depending on the pathogen, different components of the host immune system play a role in the response to an infection. Lymphocytes are important in the combat against viruses, whereas granulocytes play a key role in bacterial and fungal infections, and proliferation and activation of these immune cells is regulated by cytokines and interferons. Over the last decades it became clear that also Natural Killer (NK) cells are involved in the host response against all of these pathogens.

In the immunocompromised patient, the recovery of the immune system has a major impact on the outcome of an infectious complication [4, 5]. Therefore, there is major interest in strengthening the host response in immunocompromised patients, either by using cytokines or growth factors or by adoptive cellular therapies transfusing immune cells such as granulocytes or pathogen-specific T-cells [6–8]. To date, relatively little is known about the potential of adoptively transferred NK cells in patients with infectious complications, although the anti-cancer property of NK cells is intensively being investigated in the clinical setting. Notably, in addition to strengthen the host response against a pathogen, potential adverse effects have to be considered, such as excessive inflammation by pro-inflammatory cytokines resulting in tissue damage [9–11]. This review will focus on the antimicrobial properties of NK cells and the current standing and future perspectives of generating and using NK cells as immunotherapy in patients with infectious complications, an approach which is promising and might have an important clinical impact in the future.

### NK cell biology

**Figure 1:**
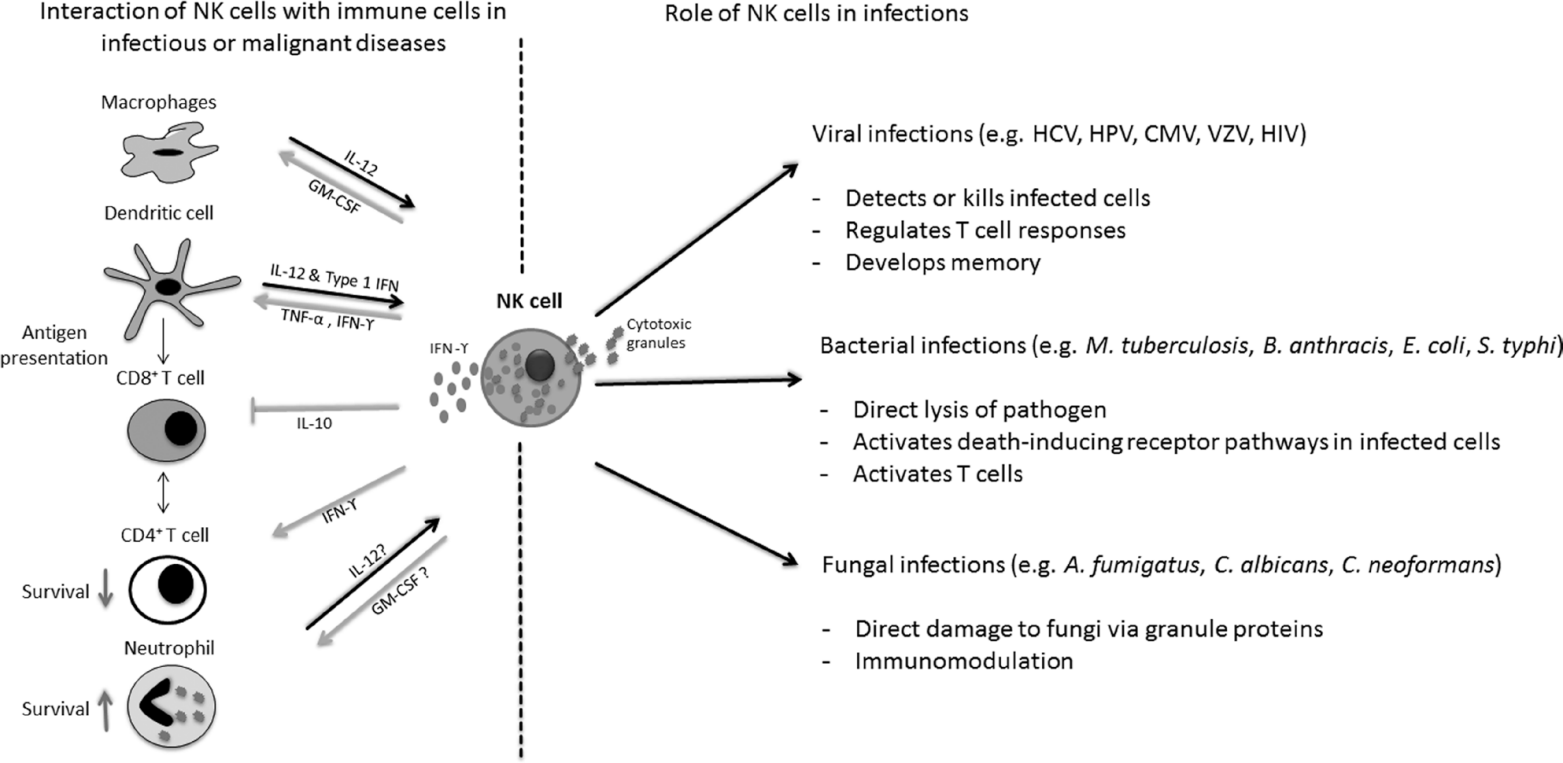
Antimicrobial activities of Natural Killer (NK) cells NK cells not only detect and damage various viral, bacterial and fungal pathogens (right side), but also modulate proliferation and activation of a variety of cells of the innate and adaptive immune system (left side). IL interleukin; IFN interferon; TNF tumor-necrosis factor; GM-CSF granulocyte-macrophage colony-stimulating factor; HCV hepatitis C virus; HPV human papilloma virus; CMV cytomegalovirus; VZV Varicella-Zoster virus; HIV human immunodeficiency virus.

Human NK cells are cytolytic innate immune cells that are defined by the expression of CD56 and by the absence of the T cell marker CD3. NK cells originate from the bone marrow, home to secondary lymphoid tissues, and represent up to 15% of peripheral blood mononuclear cells. In respect to the surface expression density of CD56 and CD16, two main subpopulations of NK cells can be distinguished, namely the cytotoxic CD56^dim^CD16^bright^ and the immune regulatory CD56^bright^CD16^dim^ subsets [12]. The activity of NK cells depends on the surface expression of several activating and inhibitory receptors that recognize MHC class-1 molecules [13–17]. Activating receptors include the natural cytotoxicity receptors NKp46, NKp44, or NKp30 and NKG2D which recognize ligands and are upregulated during cellular stress such as tumor transformation and viral infections [16, 18]. Among the inhibitory receptors, the killer-immunoglobulin-like receptors (KIRs) play an important role in NK cell alloreactivity [19]. NK cells are able to kill their target directly by cytotoxic molecules such as perforin or granzyme B, and by death receptor mediated apoptosis [20, 21]. In addition to their cytotoxic function, NK cells are able to modify the immune response of the host by secreting different chemokines, like tumor-necrosis-factor alpha (TNF-α), granulocyte-macrophage colony-stimulating factor (GM-CSF) or CCL5 (RANTES) and interferon (IFN)-γ [22–24] (Figure 1). NK cells have recently been classified closely to group 1 innate lymphoid cells (ILCs), which are characterized by the ability to produce IFN-γ, but not type 2 cytokines [25].

Current research has shown that NK cell education and differentiation plays an important role in both the direct and antibody-dependent functionality of NK cells [26, 27]. In this respect, various models have been developed to explain the process of NK cell “education” [23, 28]. In general, the expression of self-recognizing inhibitory receptors (SRIR) guides NK cell development towards fully functional mature NK cells and has been termed “licensing” process [29]. The “disarming” model describes NK cells lacking SRIR that become anergic due to chronic activation [30], and the more dynamic “rheostat model” has been introduced to describe that stronger inhibitory signaling through more SRIR interactions leads to greater functional responsiveness of NK cells [31, 32]. It has been reported that SRIR deficient NK cells can be primed to a functional state upon cytokine stimulation [29]. Of note, also uneducated NK cells may play important roles in the combat against viral infections, since SRIR-deficient NK cells strongly respond toward murine CMV [33]. In conclusion, all these results underline the value of a highly diverse NK cell repertoire with unlicensed and licensed cell subsets that interact in the defense of infectious diseases [34]. Importantly, NK cells also have the ability to shape adaptive immunity such as immunological memory [35–37], as both animal models and human studies indicate that NK cells can develop long-lasting antigen-specific memory cells [38]. These aspects of NK cell regulation are highly interesting as it demonstrates the high potential of NK cells for immuno-prophylactic and/or immunotherapeutic strategies, which may be important not only in the field of cancer therapy but also for infectious diseases [23, 39].

### Anti-cancer activity of NK cells

It is well known that NK cells play a crucial role in the defense against cancer, and that low NK cell activity is associated with an increased risk for malignancy [40, 41]. The direct cytotoxicity of human expanded NK cells against several tumor cell lines could be demonstrated in various experimental settings, for example against human U87 glioblastoma cells [42], the neuroblastoma cell line UKF-NB-3 [43], and various Ewing sarcoma and rhabdomyosarcoma cell lines [44]. While cytotoxic NK cells directly eliminate tumor cells, regulatory NK cells also play an important role in the antitumor activity by orchestrating the complex interaction of other immune cells through secreted cytokines and chemokines [20, 45, 46].

### Anti-cancer immunotherapy with NK cells – clinical aspects

A number of clinical trials investigated the immunotherapeutic anti-cancer property of NK cells in various patient populations. For example, regional arterial administration of autologous cytokine-stimulated NK cells (lymphokine-activated killer cells; LAK) in combination with daily administration of low IL-2 doses to patients with metastatic renal cell carcinoma resulted in the regression of five out of 15 treated metastases and pain relief in six out of ten patients [47]. A similar approach was used in patients with recurrent glioblastoma [48]. Although the response rate of immunotherapy was 33%, the survival of 18 months from diagnosis could not be improved. A phase I clinical trial using expanded autologous NK cells (approximately 4720-fold expansion) in patients with advanced digestive cancer demonstrated that the cells were well tolerated [49]. Despite the fact that the cells were highly lytic and a strong expression of functional markers such as NKG2D and CD16 was found, no clinical response could be observed. Safety and efficacy of multiple infusions of autologous activated and expanded NK cells in combination with anti-myeloma drugs were evaluated in a recent single-arm open-label phase I clinical trial which included five patients with relapsed or refractory multiple myeloma who had received two to seven prior lines of therapy [50]. Four of the five patients showed disease stabilization prior to the end of treatment, and two showed a 50% reduction in bone marrow infiltration and a long-term response of more than one year. The use of allogeneic NK cells for immunotherapy is currently being evaluated in different settings, such as acute lymphoblastic or myeloid leukemia (ALL and AML, respectively), but also in high-risk solid tumors (reviewed in [19]). Most of the patients receive a combined treatment which includes haploidentical HSCT and the adoptive transfer of NK cells from the same haploidentical donor followed by IL-2 application, and the overall results suggest a decrease of the relapse rate. For example, in one trial, the adoptive transfer of human haploidentical NK cells in AML patients induced complete hematologic remission in five out of 19 poor-prognosis patients [39]. In contrast to the haploidentical transplant setting, the use of allogeneic NK cells in unrelated HSCT is less clear and a matter of controversy [19]. In a phase I clinical study which evaluated allogeneic NK cells from random healthy donors in 17 patients with malignant lymphoma or advanced or recurrent solid tumors, no serious adverse event occurred [51]. This corroborates the data of other studies that immunotherapy with NK cells is usually safe and well tolerated, and only temporary side effects such as fever, weight gain, or neurotoxicity are observed [52]. Notably, in a phase II study which enrolled 20 patients with recurrent ovarian and breast cancer, one ovarian cancer patient developed tumor lysis syndrome within 6 h of the initial NK cell infusion. The timing of the event, together with the detection of NK cells in the necrotic liver specimen on autopsy, suggests that NK cells could have contributed to the tumor lysis [53].

To this end, the use of NK cells as immunotherapy against cancer is promising, but further studies are warranted in order to identify patient populations which will significantly benefit from this strategy, and to tailor pharmacological and immunological therapies to the individual patients´ characteristics.

### NK cells in the host response against infectious pathogens

There is a growing body of evidence that NK cells play a major role in the host response against various pathogens. For example, a number of studies have demonstrated that genetic mutations may lead to reduced NK cell numbers or functional NK cell impairment such as mutations affecting genes *IL2RG*, *JAK3*, and *ADA* which cause severe combined immunodeficiency syndromes [54, 55] or a mutation in the *ITGB2* gene associated with leukocyte adhesion deficiency [56]. These immunocompromised patients have an increased susceptibility to viral infections, such as infections with herpes simplex virus (HSV), Varicella Zoster virus (VZV), Cytomegalovirus (CMV), and with human papilloma virus [22, 41, 57]. However, as these patients display multiple defects of the immune system, the exact role of NK cells in the increases risk of viral infection remains unclear. An early report described a young girl who experienced a series of recurrent and severe viral infections during childhood and adolescence, including infections by multiple herpes viruses, which was thought to be the result of non-functional NK cells [58]. Other studies reported on children with altered forms of the Fc receptor for IgG type IIIA (CD16) on their NK cells, who suffered from recurrent viral infections such as infections due to HSV, Epstein-Barr virus (EBV) and VZV, respectively [59, 60]. The clinical condition of these children significantly improved with acyclovir prophylaxis. Recently, it has been shown that decidua NK cells inhibit human immunodeficiency virus (HIV)-1 infection in pregnancy [61]. Similar to the fight against cancer cells, NK cells limit viral burden not only by killing of infected cells [38], but also by modulating the cytokine milieu, which in turn influences other immune cells such as T cells. For example, NK cell derived IFN-γ is not only important for the direct non-cytopathic inhibition of the replication of the hepatitis C virus [62], but also regulates the immune responses of CD4^+^ and CD8^+^ T cells [63–65]. Importantly, recent data of animal and human studies indicate that NK can develop long-lasting antigen specific memory cells [38]. Much work has been performed on the evaluation of the importance of NK cells in the host response against influenza virus. It has become clear that the severity of influenza disease is not uniform, with a severe clinical course being associated with transient T and NK cell deficiency [66] and with specific haplotypes of killer-immunoglobulin-like receptors (KIRs) [67]. In a mouse model, infection with a high dose of influenza virus led to the impairment of cytotoxicity and IFN-γ production by spleen NK cells and to decreased virus-specific killing mediated by cytotoxic T lymphocytes. Importantly, the latter could be reversed by the adoptive transfer of spleen NK cells harvested from low-dose-infected mice [68]. During influenza infection, NK cells are activated by different mechanisms, such as by influenza nucleoprotein (NP) and matrix 1 (M1) antibodies [69], and CD16 seems to play an important role in the early activation of NK cells after vaccination against influenza [70]. A recent study demonstrated that shortly after infection with influenza virus, licensed (“functional”) NK cells serve as early innate effectors as they produce IFN-γ in inflamed parenchymal tissues and further mediate direct antiviral responses [34]. In contrast, NK cells which lack self-specific MHC-I receptors (“unlicensed” NK cells) are localized in the draining lymph nodes and help to promote activation and expansion of dendritic cells, which ultimately results in a sustained antigen-specific CD8^+^ response. In addition to the killing of virus-infected cells, NK cells provide vital cytokines for tissue regeneration, such as IL-22 [71]. However, it is important to note that in mouse models, NK cells might mediate pathology as the depletion of NK cells *in vivo* reduced mortality from influenza infection, whereas the adoptive transfer of NK cells from influenza-infected lung, but not from uninfected lung resulted in increased mortality in influenza-infected mice, probably due to a deleterious NK cell-dependent alteration of T cell responses [72].

Compared to the antiviral activity of NK cells, considerably less data are available for the interaction of NK cells with bacteria and fungi. NK cells exhibit direct activity against a variety of Gram-positive and Gram-negative bacteria such as *Mycobacterium tuberculosis, Bacillus anthracis*, *Escherichia coli* or *Salmonella typhi* by the secretion of the soluble molecules perforin and granulysin [73–76]. In addition, NK cells have an antibacterial effect against intracellular bacterial pathogens by using death inducing receptor pathways such as Fas-FasL and TNF-related apoptosis-inducing ligand (TRAIL) pathways [77, 78], which ultimately induce caspase-dependent apoptosis of the target cell [21, 77, 79]. The important role of the antibacterial activity of NK cells *in vivo* is demonstrated by animal models which showed higher survival rates and lower bacterial titers during infection with *Shigella flexneri* in mice lacking B and T cells but having NK cells as compared to mice which lack all three cell types [80].

Similar to the antibacterial activity, NK cells exhibit *in vitro* antifungal activity against a number of pathogenic fungi such as *Aspergillus fumigatus*, *Candida albicans*, *Cryptococcus neoformans*, or different species of mucormycetes [81–86]. Cytotoxic molecules including NK cell derived perforin seem to be important in the antifungal activity. In addition, upon stimulation by fungi, NK cells release a number of cytokines, which modulate both innate and adaptive immune responses [87]. The *in vitro* data of the antifungal activity of NK cells are supported by observations made in animal studies. For example, it has been shown that NK cells proliferate in mice experimentally infected with *Aspergillus niger*, and this proliferation was associated with an inhibition of the fungal growth [88]. Antibody mediated depletion of NK cells in mice inoculated with *C. neoformans* [89] resulted in a significant higher fungal burden in the lungs as compared to untreated controls, corroborating studies in NK-depleted mice which revealed the pivotal role of NK cells in the host response against *A. fumigatus*, *C. albicans*, and *Histoplasma capsulatum* [90–95].

Although these data clearly demonstrate that NK cells exhibit important activities in the host immune response to different viral, bacterial and fungal pathogens, many questions have to be resolved. For example, further studies have to evaluate how and to which extent a pathogen may exert an immunosuppressive effect on NK cells as well as on other cells of the immune system, which has been shown for bacteria such as *Pseudomonas aeruginosa* and for fungi such as *A. fumigatus*. This knowledge may be important in the clinical setting for specifically strengthening the host response during infection.

### The strategy of adoptive immunotherapy in infectious complications

Both severity and duration of a defect in the immune system are associated with risk and outcome of an infectious complication [4]. Therefore, since the 1970s, there was great interest to improve the prognosis of persistently neutropenic patients suffering from severe infections with the administration of granulocytes, and the availability of recombinant hematopoietic growth factors such as granulocyte-colony stimulating factor (G-CSF) extended the use of this strategy even into the prophylactic setting (“adoptive immunotherapy”) [96]. A prospective, non-randomized study evaluated transfusions of granulocytes in order to control acute life-threatening infections or to prevent recurrence of severe fungal infections during HSCT or intensive chemotherapy [6]. Granulocyte transfusions achieved control in 82% of acute life-threatening infections, and no single reactivation of a previous infection occurred with prophylactically administered granulocytes, whereas a recent randomized study failed to demonstrate a significant benefit of this strategy [97]. In patients suffering from invasive fungal infections, a recent review of available data did not find strong evidence of a benefit of granulocyte transfusions, but it is to hope that ongoing randomized controlled studies such as the GRANITE study (German Clinical Trials Register number DRKS00000218) will provide helpful results [98]. Notably, granulocyte transfusions are often associated with febrile transfusion reactions and pulmonary complications, including transfusion-related acute lung injury (TRALI), and a monocenter retrospective analysis of 128 patients with a hematological malignancy, prolonged neutropenia and invasive aspergillosis suggested that patients receiving granulocyte transfusions had a worse outcome [99]. Another approach which aims to reconstitute the long-lasting impairment of cellular immunity of allogeneic HSCT recipients became possible with the development of techniques to isolate and to generate pathogen-specific T cells. Infusion of CMV-specific T cell clones largely prevented CMV reactivation and reduced CMV mortality [7, 100], and studies reported on a clinical benefit for adoptively transferred T cells specific against adenovirus [101] and Epstein-Barr-virus (EBV) [102], respectively. Similarly, a proof-of principle study showed that the adoptive transfer of pathogen-specific *Aspergillus* CD4^+^ T cells resulted in a rapid decline of *Aspergillus* antigen in the blood, and more importantly, 9 of 10 patients cleared invasive aspergillosis [7]. In contrast to most studies which use donor-derived pathogen specific T cells, a recent trial reported on the successful use of “off the shelf” T cells generated from eligible third-party donors against a variety of viruses such as BK virus, human herpesvirus 6, adenovirus or EBV [103].

Although immunotherapy with adoptively transferred pathogen specific T cells seems to be a promising strategy, the use of T cells may be associated with the risk of graft-versus-host disease (GvHD) [104].

### NK cells as potential immunotherapeutic agent in infectious complications

As compared to studies investigating NK cells as immunotherapeutic tool in patients with an underlying malignancy, relatively little is known regarding the *in vivo* effect of adoptively transferred NK cells into a host suffering from an infectious complication. In *Aspergillus* infected mice, the depletion of NK cells resulted in a higher fungal load and lower survival, whereas the transfer of activated NK cells to these mice led to greater pathogen clearance from the lungs [93]. Similarly, cyclophosphamide pretreated mice suffering from cryptococcosis showed an enhanced clearance of the fungus when they had received an NK cell-enriched graft as compared to mice which had received an NK cell-depleted graft [105, 106]. Although these results suggest that adoptively transferred NK cells may be a potential tool in patients suffering from fungal infections, no study to date has proven this concept in the clinical setting. Importantly, there may be a major difference of the adoptive transfer of NK cells between immunocompromised and immunocompetent patients. Whereas safety data in immunocompromised patients with cancer are promising [107, 108], results on the adoptive transfer of NK cells into immunocompetent human individuals are lacking. However, it has been demonstrated that NK cells infused in mice with polymicrobial intra-abdominal bacterial infection contributed to an excessive induction of pro-inflammatory cytokines which ultimately resulted in a lethal septic shock [10, 11, 109–111]. Another study in the murine model demonstrated that infection by *Listeria monocytogenes* resulted in excessive IFN-γ production by CD27^+^ NK cells, which in turn, impaired innate anti-bacterial host defenses by inducing down-regulation of CXCR2 on granulocytes and thus inhibiting the recruitment of granulocytes at the site of infection [112]. However, the mice could be rescued by antibodies blocking CD27 signaling or by depleting IFN-γ. Therefore, further studies have to better define the preconditions in which the adoptive transfer of NK cells may be beneficial or harmful, such as the degree of the host´s immune impairment or the pathogen(s) causing an infection, as well as the optimal schedule and dosage of adoptively transferred NK cells in each setting. On the other hand, as compared to pathogen-specific T cells which have already been evaluated in the clinical setting, NK cells may be of advantage since they are active against a broad range of pathogens including viruses, bacteria, or fungi.

### Generation of high numbers of functionally active NK cells for adoptive immunotherapy

When employing NK cells to fight against a malignancy or to combat infectious complications, only functional active NK cells administered at a sufficient effector-to-target (E:T) ratio might result in a beneficial effect. Studies addressing both mouse and human NK cell immunity have shown that NK cells are a heterogeneous population consisting of not only phenotypically but also functionally distinct subsets, and cytokine-stimulation induces significant changes not only in proliferation, maturation status and finally in the subset composition of the *ex vivo* expanded NK cell product [45, 113]. Therefore, knowledge on mouse NK cells cannot directly be transferred to the human system, and expansion and activation studies are mainly performed on human NK cell preparations from peripheral blood or umbilical cord blood leading to first preclinical data evaluating the cytotoxic capacity of the final NK cell product *in vitro* and *in vivo* in xenograft NSG (NOD scid gamma) mouse models against various human tumors [114].

Most clinical trials with NK cells in the autologous or allogeneic setting administer *ex vivo* expanded NK cells [115, 116]. Of note, the expansion of NK cells usually needs several days to weeks, depending on the protocols used [114, 117]. In order to improve the rapid expansion of isolated NK cells, cytokines like IL-2 have been investigated, which can be added either alone or in combination with an anti-CD3 antibody or additional cytokines such as IL-15 and IL-21 [117–120]. Although the exact role of anti-CD3 antibodies is unclear, it has been suggested that OKT-3 leads to a profound outgrowth of NK cells, which is probably due to the activation of T cells [114]. Other studies suggest that co-culture of NK cells with stimulatory cells such as EBV-transformed lymphoblastoid cells or a Wilms tumor-derived cell line also enhances the proliferation of NK cells [121, 122]. In an elegant approach, HLA-negative K562 cells were genetically modified to express membrane-bound IL-15 and 4-1 BB Ligand (4-1BBL), which specifically activate NK cells and promote their proliferation and survival, and this strategy resulted in a dramatic enhancement of NK cell expansion and activation [123, 124]. After further improvement, this method has been adapted to large-scale Good Manufacturing Practices (GMP) conditions [121, 125]. In addition to autologous or allogeneic NK cells, it may be possible to differentiate NK cells *in vitro* from umbilical cord blood CD34^+^ cells, which is currently being tested in two clinical trials (NCT01619761 and NCT01729091) [126].

In addition to proliferation, cytokine stimulation may also improve the functional activity of NK cells. For example, when NK cells are resting, lytic granules are distributed randomly or diffuse, whereas after exposure to IL-2, granules congregate to the microtubule-organizing center [127]. Thus, IL-2 stimulation not only activates NK cells, but also accelerates their transition into NK cells which are ready to exhibit their cytotoxic function. Another study demonstrated that exposure of murine NK cells to IL-12, IL-15 and IL-18 resulted in a sustained effector function of NK cells *in vivo* [128]. Only IL-12/15/18-preactivated NK cells, but not naïve, IL-15- nor IL-2-pretreated NK cells, respectively, reduced the growth of tumors when mice were concomitantly irradiated. Similarly, prestimulation with a cytokine cocktail including IL-12, IL-15, and IL-18 resulted in enhanced IFN-γ production of human NK cells after restimulation with K562 leukemia cells or with these cytokines [129]. Even if there are known differences in the biology of murine and human NK cells, this observation suggests that both murine and human NK cells receive functional memory-like properties after cytokine activation, which may provide a novel rationale for integrating cytokine preactivation into NK cell immunotherapeutic strategies. However, at the same time, the use of cytokines may alter the phenotype of the NK cell and result in a potential loss of responsiveness to some stimuli [130, 131]. For example, IL-2 stimulation of NK cells decreased CD16 [132], and NK cell activation by intramuscular influenza vaccination and HIV-positive plasma induced a matrix metalloproteinase-mediated cleavage of cell surface CD16, whereas inhibition of CD16 shedding potentiated NK cell cytotoxic function [70, 133].

In addition to the exposure to various cytokines, improvement of NK cell activity can be achieved by the manipulation of NK cell receptors. For example, NK cell cytotoxicity against tumor targets could be improved by a retroviral transduction of a receptor termed NKG2D-DAP10-CD3ζ that is composed of NKG2D plus two signaling molecules, DAP10 and CD3ζ [134]. These modified NK cells exhibit higher killing activity against a number of ALL cell lines such as CEM-C7 or MOLT-4, and against solid tumor-derived cell lines such as the human prostate adenocarcinoma cell line LNCaP or the hepatocellular carcinoma cell line HepG2 [134]. Importantly, the increased activity of the modified cells could also be demonstrated *in vivo*, since in immunodeficient mice, NKG2D-DAP10-CD3ζ-transduced NK cells killed osteosarcoma or hepatocellular carcinoma cells more effectively compared to mock-transduced NK cells [134, 135]. However, soluble NKG2D ligands (sNKG2DL) such as sMICA/B or sULBP2 are able to impair NK cell activity as it has been demonstrated in the setting of various tumors and of HIV [43, 136]. Naïve HIV-positive patients display increased plasma levels of sMICA and reduced NKD2D expression on NK cells, and sNKG2DLs impair NKG2D-mediated cytotoxicity of NK cells [136]. Interestingly, highly active antiretroviral therapy (HAART) resulted in the drop of sNKD2DL and recovery of NKD2D expression.

Another interesting approach is the transduction of chimeric antigen receptors (CARs) into immune cells in order to improve their activity. CARs are engineered receptors with the ability to bind to specific antigens which are expressed on the surface of tumor cells or on the surface of a pathogen. The strategy of CARs in patients’ T cells has recently been reported, which resulted in impressive regression of B cell malignancies, and the promising results of first clinical studies on CAR-T cells have recently been reported [137–140]. Of note, CAR constructs expressed in the NK cell line NK92 [141], primary NK cells [137, 142] or cord blood derived NK cells [143] have demonstrated efficient killing in preclinical settings [144]. Two clinical trials testing haploidentical donor-derived CAR NK cells for targeting of refractory CD19^+^ ALL with a second-generation anti-CD19 CAR that incorporates the 4-1BB costimulatory domain are currently recruiting patients (NCT00995137 from St. Jude and NCT01974479 from The National University Health System, Singapore) (for review see [145]).

In contrast, studies on NK cells with modified receptors or CAR-NK cells against infections are lacking, and challenges of this strategy include the relatively long time to generate these cells and the complex antigenic properties of many pathogens, which is seen in particular in fungi [146].

The functional activity of NK cells can also be enhanced by the forced expression of the high-affinity CD16-158V (HA-CD16) Fc receptor, for which the minority of patients is homozygous [147, 148]. Several groups demonstrated that engraftment of HA-CD16 on primary NK cells from donors which express a low-affinity CD16-158F/F (LA-CD16) Fc receptor [149] or on the NK-92 cell line [150] significantly increases the antibody-dependent cell-mediated cytotoxicity (ADCC) against target cells coated with rituximab, an antibody directed against CD20, of which the Fc portion mediates both antibody-dependent cell-mediated and complement-dependent cytotoxicity. Rituximab is used as therapeutic compound in many patients suffering from B cell non-Hodgkin lymphoma.

The genetic disruption of NK cell inhibitory receptors such as KIR or NKG2A via inhibiting antibodies or via shRNA silencing may also be used to overcome tumor evasion mechanisms related to MHC I expression. In this regard, in tumor bearing mice, NKG2A-silenced cells of the NKL cell line revealed enhanced killing activity of 721.221 HLA-E expressing EBV-LCL tumor cells [151–153]. Similarly, in the setting of an infection, blocking the interaction between NK cell inhibitory receptors [e.g., CD159a (NKG2A), CD158a (KIR2DL1), and 158b (KIR2DL2)] and MHC class I molecules (e.g., HLA-C and HLA-E) on HIV-infected autologous tumor cells resulted in a drastic increase in killing of anti-gp120-coated HIV-infected cells by NK cells [154].

### Immunotherapy in infectious complications using NK cells from a cell bank

**Figure 2:**
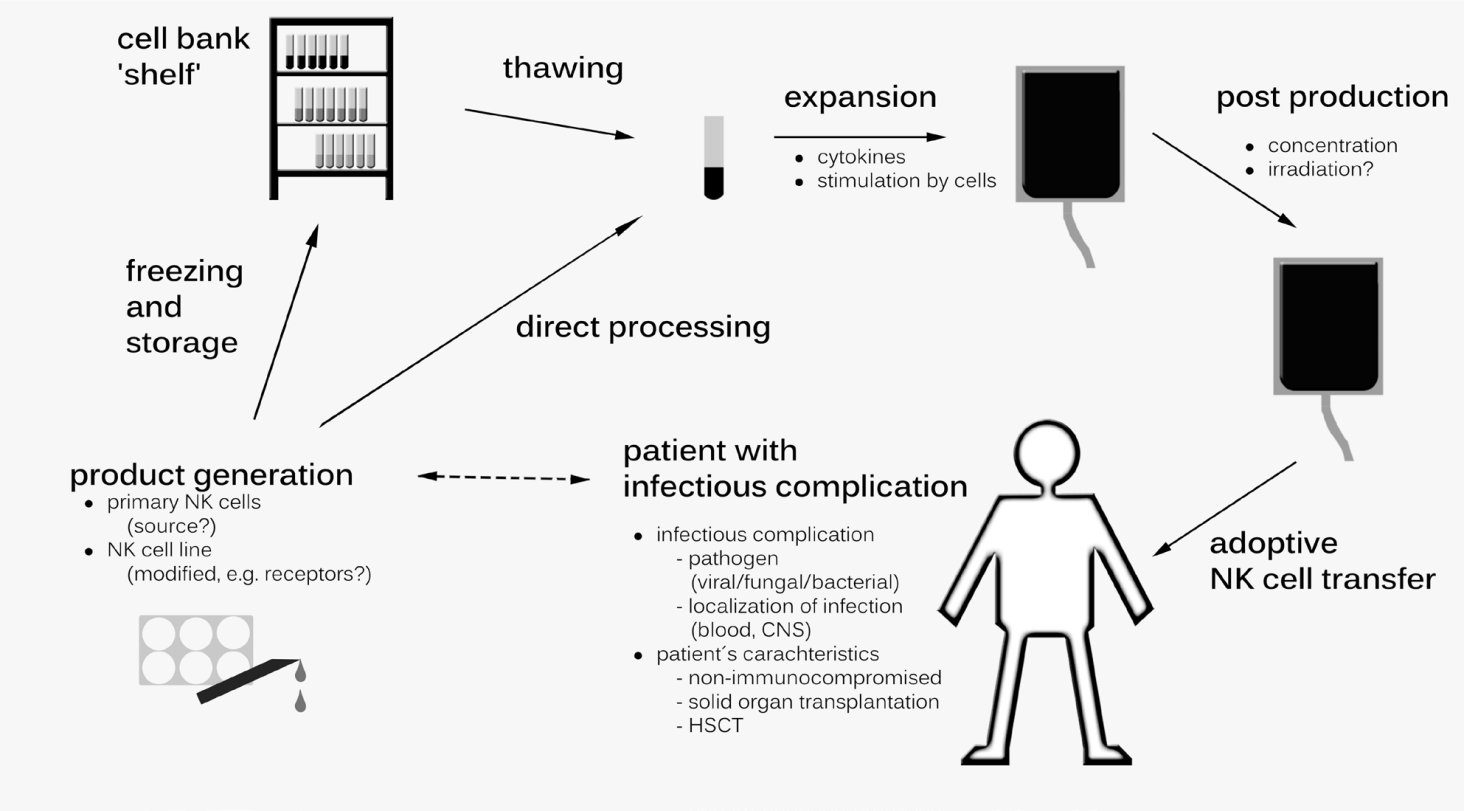
Potential strategies of generating Natural Killer (NK) cells as an immunotherapeutic tool for patients suffering from infectious complications Both the infectious complication (e.g., pathogen, localization) and the patient´s characteristic influence the generation of the NK product (e.g., primary cells, NK cell line), which can be directly processed or be frozen and stored. CNS, central nervous system; HSCT, hematopoetic stem cell transplantation.

In particular in immunocompromised patients, infections often have a sudden onset with a rapid clinical course. Therefore, an immunotherapeutic tool in this setting has to be quickly available, which might be different to the setting of immunotherapy used against an underlying malignancy. In other words, the timely access to a suitable NK cell product is crucial when planning clinical studies evaluating NK cells in patients with viral, bacterial, or fungal infections (Figure 2). In this respect, NK cell products which are standardized, well characterized and cryopreserved would be ideal and open new perspectives in this emerging field. However, the long-term storage of NK cell products remains controversial. Whereas it has been demonstrated that NK cells maintain their cytotoxic activity against the leukemia cell line K562 after cryopreservation [126, 155], standard methods of cryopreservation seem to have a negative impact on cell expansion *in vivo* [156]. Interestingly, in one clinical trial NK cells were expanded upon medical need from aliquots of individual cryopreserved leukapharesis cryopreserved peripheral blood mononuclear cells [157].

### NK cell lines as the source for NK cell immunotherapy

Having identified the challenges in derivation, activation and expansion of NK cells directly from patients, NK cell lines may be considered as an ideal source for cell-based immunotherapy. Advantages using NK cell lines for immunotherapy would include 1) the possibility to establish a master cell bank, and 2) the fact that the cell source would be extremely well standardized and characterized. As one example, the permanent NK-92 cell line is cryopreserved in GMP-compliant master cell bank, from which it can be easily and reproducibly expanded [158]. The cell line exhibits cytotoxicity against a broad spectrum of tumor targets *in vitro* such as various leukemia, lymphoma and myeloma cell lines as well as against primary leukemic blasts [159–162]. When used as immunotherapy against cancer, preliminary data demonstrated the safety and tolerability of NK-92 cells in the clinical setting [163, 164]. A number of groups are exploring the use of CAR-modification to enhance the antitumor activity of these cells in preclinical and clinical studies [165, 166] (for review see: [145, 167]).

However, to date, relatively little is known about the activity of the NK-92 cell line against infectious pathogens. NK-92 cells reveal *in vitro* activity against EBV-infected B lymphocytes and the supernatant collected from NK-92 cells inhibits HIV replication in PBMCs from HIV-infected subjects in a dose-dependent manner [168]. In contrast, hepatitis B virus (HBV) antigens HBsAg and HBeAg have a direct negative impact on NK-92 cell activation, cytokine production and cytotoxic granule release [169]. In addition, the lack of toll-like receptor (TLR) 4 and CD16 (Fc receptors FcγRIIIa and FcγRIIIb) might be a relevant limitation of this cell line regarding the antimicrobial activity, since, for example, TLR4 is responsible for the detection of FimH, which is present of fimbria of some bacteria [170]. Other drawbacks in the clinical use of NK-92 cells include the fact that the NK-92 cell line is derived from an NK cell lymphoma and therefore has the potential risk for uncontrolled proliferation, in particular if cells are resistant or not fully hit by prior irradiation. In turn, irradiation, which is mandatory prior to infusion, severely limits the survival and function of the transferred cells. Since NK-92 cells are dependent on IL-2, the repeated IL-2 injections raise concerns regarding toxicity [171]. Whether this potential toxicity can be abrogated by genetic modification of the cells leading to constitutive expression of IL-2 and resulting in auto-activated and auto-proliferating cells is unclear to date [148, 149]. Of note, in a promising preclinical study, transduction of clinically applicable NK-92 cells with lentiviral vectors encoding human IL-15 resulted in predominantly intracellular expression of the cytokine, proliferation and cytotoxicity of the producer cells in the absence of IL-2 [172].

Based on these experiences, further studies evaluating the activity of the NK-92 cell line against viral, bacterial, and fungal pathogens are urgently needed.

It is important to mention that in addition to the NK-92 cell line, there are other cell lines which potentially could be used as adoptive NK cell-based immunotherapy, both in patients with cancer and in patients with infectious complications. For example, the KHYG-1 cell line derived from NK leukemia has superior cytotoxicity compared to NK-92 cells [173]. In addition, irradiation of these cells does not abrogate their cytotoxicity towards tumor targets. Similarly, the cell line NKL, which is biologically and functionally very similar to primary NK cells, exhibits enhanced cytotoxicity against certain tumor cells as compared to NK-92 [174]. Again, the antimicrobial activity of these cells lines is unclear and needs further evaluation.

## CONCLUSIONS AND FUTURE PERSPECTIVES

Whereas the anticancer effect of NK cells is currently investigated in multiple clinical trials, little is known about the potential of adoptively transferred NK cells in patients suffering from infectious complications. *In vitro* data clearly demonstrate that NK cells are active against viral, bacterial, and fungal pathogens, and animal studies suggest that NK cells could be a promising tool in the antimicrobial immunotherapy. Current investigation focuses on the optimal and rapid generation of high numbers of functionally active NK cells and includes strategies such as genetic modification of the cell and its receptors and the use of NK cell lines for treatment of hemato-oncological diseases. However, before testing adoptively transferred NK cells in the clinical setting of infectious complications, a number of questions have to be resolved. For example, it is well known that activation of the host immune system (e.g., by the release of pro-inflammatory cytokines) is necessary in order to successfully fight the pathogen, but, at the same time, might cause severe complications, as it was documented in patients with pulmonary aspergillosis who received granulocyte transfusions [99]. Therefore, both the patient population (immunocompromised versus immunocompetent) and the optimal time point of the adoptive transfer of NK cells are unknown to date, or, in other words, it is unclear when and whom adoptively transferred NK cells will help to overcome an infectious complication or will ultimately harm. Based on the promising results in animal studies and due to the facts that HSCT recipients suffering from fungal infections lack a sufficient immune response and outcome of this patient population is extremely poor, first clinical trials might focus on invasive fungal disease in this patient population. Although it will be necessary to fully characterize the optimal patient population, the best time point of therapy as well as the best approach to generate NK cells for immunotherapy in infectious complication, this strategy might become important in this setting, in particular since antimicrobial compounds have limited activity and we witness emerging resistance of pathogens all over the world.
